# Evaluation of Short-Season Soybean Genotypes for Resistance and Partial Resistance to *Phytophthora sojae*

**DOI:** 10.3390/ijms24076027

**Published:** 2023-03-23

**Authors:** Shengfu He, Xiran Wang, Xiaohui Sun, Yuxin Zhao, Simei Chen, Ming Zhao, Junjiang Wu, Xiaoyu Chen, Chuanzhong Zhang, Xin Fang, Yan Sun, Bo Song, Shanshan Liu, Yaguang Liu, Pengfei Xu, Shuzhen Zhang

**Affiliations:** 1 Soybean Research Institute, Key Laboratory of Soybean Biology of Chinese Education Ministry, Northeast Agricultural University, Harbin 150030, China; 2 Soybean Research Institute, Key Laboratory of Soybean Cultivation of Ministry of Agriculture, Heilongjiang Academy of Agricultural Sciences, Harbin 150086, China

**Keywords:** short-season soybean, resistance, partial resistance, *P. sojae*

## Abstract

Phytophthora root and stem rot caused by *Phytophthora sojae* Kaufmann and Gerdemann is a soil-borne disease severely affecting soybean production worldwide. Losses caused by *P. sojae* can be controlled by both major genes and quantitative trait locus. Here, we tested 112 short-season soybean cultivars from Northeast China for resistance to *P. sojae*. A total of 58 germplasms were resistant to 7–11 *P. sojae* strains. Among these, Mengdou 28 and Kejiao 10-262 may harbor either *Rps3a* or multiple *Rps* genes conferring resistance to *P. sojae*. The remaining 110 germplasms produced 91 reaction types and may contain new resistance genes or gene combinations. Partial resistance evaluation using the inoculum layer method revealed that 34 soybean germplasms had high partial resistance, with a mean disease index lower than 30. Combining the results of resistance and partial resistance analyses, we identified 35 excellent germplasm resources as potential elite materials for resistance and tolerance in future breeding programs. In addition, we compared the radicle inoculation method with the inoculum layer method to screen for partial resistance to *P. sojae*. Our results demonstrate that the radicle inoculation method could potentially replace the inoculum layer method to identify partial resistance against *P. sojae*, and further verification with larger samples is required in the future.

## 1. Introduction

Phytophthora root and stem rot (PRSR) caused by *Phytophthora sojae* Kaufmann and Gerdemann is a destructive disease of soybean (*Glycine max* (L.) Merr.) worldwide [1]. *P. sojae* can infect soybean at various plant growth stages, especially in low-lying fields with continuous cropping, heavy soil, and poor drainage [2]. Infection results in seed rot, seedlings damping off, root and stem rot, and even soybean plant death [3]. This disease severely limits soybean production and causes economic losses of up to USD 1–2 billion annually [4].

It has been clear that the pathogenetic variation of *P. sojae* has been complex since it was first described [5], and to date, at least 55 races have been reported [6,7,8,9]. Although several measures, such as seed fungicides, application of calcium-containing compounds, and soil drainage condition improvement, etc. have been reported to mitigate the damage of *P. sojae* [2,10,11], the deployment of resistant cultivars remains the most environmentally friendly and effective strategy to limit losses caused by the disease.

Single dominant resistance genes to *P. sojae* (*Rps* genes), also known as race-specific resistance genes, have been used extensively in soybean to manage *P. sojae* [12]. Since the first identification of *Rps1* [13], more than 35 *Rps* genes/alleles have been mapped to nine chromosomes, including the newly discovered *Rps13* [14]. Based on the typical gene-to-gene theory, the effectiveness of each *Rps* gene depends on the presence of the corresponding avirulence gene (*Avr*) in *P. sojae* [4]. However, due to the emergence of new virulent races in response to selection pressure exerted by the continuous use of specific resistant cultivars, the exploitation of these *Rps* genes has often been short-lived, as their effectiveness is limited to 8–15 years [15].

Partial resistance, also known as field tolerance, is a highly heritable quantitative trait controlled by multiple genes such as *Rps1a*, *Rps1k, and Rps3a*. It does not exert selection pressure on pathogens and produces more durable resistance to *P. sojae* [12]. Currently, the focus of breeding research is on conferring partial resistance in soybean. For example, Schneider et al. [16] evaluated the resistance of 1395 plant introductions (PIs) to two highly virulent *P. sojae* isolates and screened several QTLs for partial resistance to *P. sojae*. Meanwhile, some soybean germplasm with high partial resistance were also selected as candidate parental resources [17,18]. This strategy can result in longer-lasting defense response and, to a certain extent, attenuate the incidence of disease in soybean plants following infection by *P. sojae* and thereby lessen any impact on soybean yield [19,20]. Combining partial resistance and single-gene resistance is essential to improve broad-spectrum resistance in soybean and necessitates screening for new resistance sources.

As soybean is native to China, the country possesses abundant germplasm resources. Northeastern provinces, including Heilongjiang, Inner Mongolia, Liaoning, and Jilin, are the main regions of soybean cultivation, accounting for up to 50% of the total cultivated area and yield of soybean in China, where most of the short-season soybean is grown. Since PRR was first reported in Heilongjiang Province in 1989 [21], it has become a major disease in most soybean-producing regions in China [22,23,24,25,26,27]. The virulence structure of *P. sojae* is complex, with different dominant virulence types of *P. sojae* in different regions [23]. Therefore, it is imperative to evaluate soybean germplasm resources for resistance to a range of virulence types of *P. sojae* and identify their resistance genotypes to obtain effective disease-resistant cultivars and new sources of disease resistance.

The objectives of the present study were to assess resistance and partial resistance to *P. sojae* races in early-maturing soybean cultivars grown in the short-season regions of China. In addition, we aimed to identify candidate excellent soybean germplasm resources that contain new disease resistance genes or multi-resistance gene combinations and identify candidate parental lines for PRR resistance breeding.

## 2. Results

### 2.1. Resistance of the Soybean Germplasm Resources

**Figure 1:**
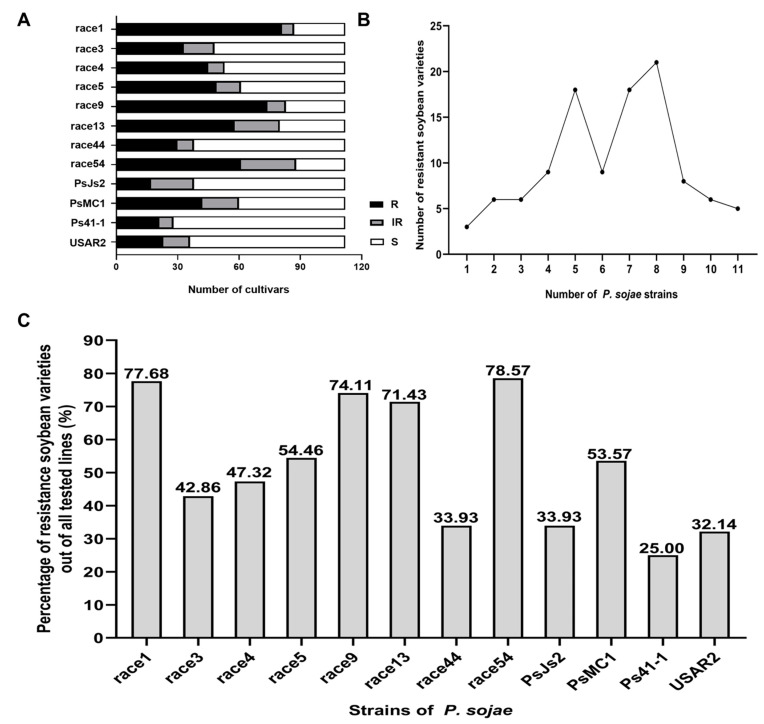
Resistance analysis of 112 soybean cultivars to 12 *P. sojae* strains. (**A**), The number of soybean cultivars showing resistance, intermediate reactions, and susceptibility against different *P. sojae* strains. R = resistance, IR = intermediate resistance, S = susceptible. (**B**), The number of germplasm resistance to different numbers of *P. sojae* strains in R + IR levels. (**C**), The percentage of germplasm resistance to different *P. sojae* strains.

We first used hypocotyl wound inoculation to identify resistance to 12 *P. sojae* strains on 112 soybean cultivars from Northeastern China. Five days after inoculation, the cultivar responses to each race were classified as resistant, intermediate, and susceptible reactions based on mortality levels of ≤30, 30–70, and ≥70%. Figure 1A shows the number of soybean cultivars showing resistance, intermediate reactions, and susceptibility against different *P. sojae* strains. The results indicate that resistance to *P. sojae* races was relatively common in the tested soybean cultivars (lines) from Northeastern China. Figure 1B shows the number of soybean germplasms resistant to different numbers of strains. Collectively, 110 germplasms were resistant to 1–11 *P. sojae* strains, accounting for 98.21% of the tested material. The percentage of cultivars with resistant and intermediate reactions (combined) to races 1, 3, 4, 5, 9, 13, 44, 54, PsJs2, PsMC1, Ps41-1, and USAR2 is shown in Figure 1C. The highest percentage of accessions with resistant and intermediate reactions was obtained in response to race 54, followed by races 1, 9, 13, 5, PsMC1, 4, 3, PsJs2, 44, USAR2, and Ps41-1. Only 25% of the total accessions were resistant to race Ps41-1.

Among the 112 soybean cultivars, a total of 58 germplasms were resistant to 7–11 *P. sojae* strains. Dengke 4, Mengdou 28, Kejiao 10-262, Heinong 57, and Suinong 35 were resistant to 11 *P. sojae* strains; Jiyu 35, Henong 75, Dengke 3, Beidou 48, Kejiao 07-584, and Suinong 32 were resistant to 10 *P. sojae* strains; Dengke 1, Suinong 33, Suinong 36, Henong 67, Suinong 29, Fengshou 23, Neidou 4, and Mengdou 15 were resistant to 9 *P. sojae* strains; 21 germplasms including Mengdou 11, Mengdou 37, and Mengdou 38 were resistant to 8 *P. sojae* strains; 18 germplasms including Dengke 6, Mengdou 26, and Mengdou 34 were resistant to 7 *P. sojae* strains. Except for Suinong 28 and Henong 60, which were not resistant to 12 *P. sojae* strains, the remaining 52 germplasms, including Heihe 22, Heinong 67, and Dengke 5, were resistant to 1-6 *P. sojae* strains.

Gene postulation of the above resistance results yielded 92 resistance response types (Table S1). Mengdou 28 and Kejiao 10-262 exhibited RRRRRRRRSRRR for race1, race3, race4, race5, race9, race13, race44, race54, PsJs2, PsMC1, Ps41-1, and USAR2, and may possess either the *Rps3a* or multiple resistance *Rps* gene *Rps*1a + *Rps*3a, *Rps*1a + *Rps*1b, *Rps*1b + *Rps*1c, *Rps*1b + *Rps*1d, *Rps*1b + *Rps*3a, *Rps*1b + *Rps*6, *Rps*1c + *Rps*1d, *Rps*1c + *Rps*3a, *Rps*1d + *Rps*1k, *Rps*1d + *Rps*3a, *Rps*1d + *Rps*6 to *P. sojae*. Furthermore, 110 germplasms had unspecified genotypes, yielding 91 reaction types that differed both from lines containing a single known disease resistance gene and from those with a combination of two known disease resistance genes, thus potentially containing new disease resistance genes or gene combinations.

### 2.2. Partial Resistance of the Soybean Germplasm Resources

**Figure 2:**
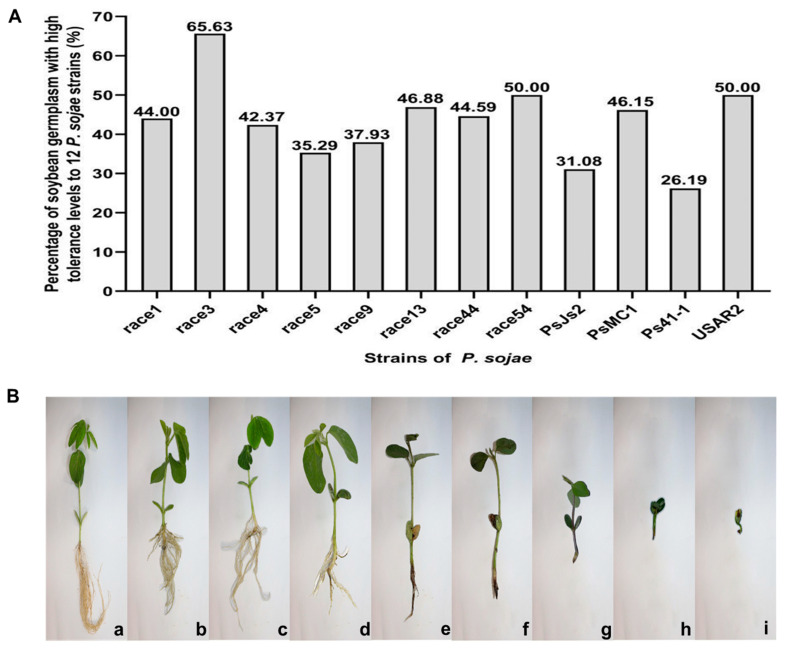
Partial resistance of the soybean germplasm resources. (**A**) Percentage of soybean germplasm with high tolerance levels to 12 *P. sojae* strains. (**B**) The reaction of roots after inoculation with *P. sojae* strain using inoculum layer method. a—The roots are not rotted, and the plant grows normally (denoted as 1); b—The roots of the plant are slightly rotted (denoted as 2); c—The roots of the plant are 1/3 rotted (denoted as 3); d—The roots of the plant are 2/3 rotted (denoted as 4); e—All roots are rotted, 10% of plants die (denoted as 5); f—50% of the plants died, and the plants were stunted (denoted as 6); g—75% of plants die, severe development of plants (denoted as 7); h—90% of plants die (denoted as 8); i—All plants die (denoted as 9).

Once a germplasm was identified as susceptible to *P. sojae* strains from the hypocotyl inoculation test, it was evaluated for partial resistance to the strains using the inoculum layer method. The scoring range of disease index is 0–100, where the mean disease index ≤ 30 is a highly tolerant germplasm. The results of disease resistance in germplasms inoculated with different strains are shown in Table S2. Of all tested cultivars (lines), 11 show a high tolerance to race 1, 42 to race 3, 25 to race 4, 18 to race 5, 11 to race 9, 15 to race 13, 33 to race 44, 12 to race 54, 23 to PsJS2, 24 to PsMC1, 22 to Ps41-1, and 38 to USAR2. The percentage of highly resistant germplasm in the test material ranged from 26.19% to 65.63% (Figure 2). Moreover, the average value of plant disease index in 34 soybean germplasms, including Heinong 51, Hefeng 46, Hefeng 52, Suinong 27, Suinong 29, Suinong 35, Suinong 38, Suizhongzuo 40, Kejiao 10–262, Beidou 42, Heihe 4, Heihe 29, Heihe 35, Heihe 43, Heihe 48, Heihe 52, Mengdou 9, Mengdou 11, Mengdou 12, Mengdou 13, Mengdou 14, Mengdou 28, Mengdou 32, Mengdou 33, Mengdou 35, Mengdou 37, Mengdou 38, Dengke 1, Dengke 4, Dengke 6, Dengke 9, Dengke 10, Kenfeng 16, and Jiyu 35 for disease tolerance identification was lower than 30. Among these 34 highly tolerant soybean germplasms, a total of 25 germplasms were resistant to 7–11 *P. sojae* strains in previous disease resistance identification and could be used as elite resistance and tolerance materials for breeding in the future. The other nine soybean germplasms were resistant to two to six *P. sojae* strains and can also be introduced into the genetic background of highly resistant cultivars for breeding and application. These results indicate that the Northeast region is rich in disease-tolerant resources and that these highly tolerant germplasms can provide excellent parents and carriers of superior genes for breeding soybean lines resistant to *P. sojae* in China.

### 2.3. Acquisition of Germplasm Resources with Resistance and Partial Resistance

The results of comprehensive resistance and partial resistance identification showed that Mengdou 28, Kejiao 10–262, Suinong 35, and Dengke 4 were resistant to 11 *P. sojae* strains, and the plant response was high disease resistance when partial resistance was identified; Heinong 57 was resistant to 11 *P. sojae* strains and was a better multi-resistant germplasm, although it was not resistant when inoculated with race 9; Jiyu 35 was resistant to 10 *P. sojae* strains, and the plant response was high disease resistance when partial resistance was identified; 29 soybean germplasms, including Heinong 51, Hefeng 46, and Suinong 27, were resistant to 2–9 strains of *P. sojae* strains, and the results of partial resistance identification showed high disease resistance (Table 1). The above 35 soybean germplasms were identified as suitably resistant and tolerant materials and could be used as excellent parents for breeding against PRR in China.

### 2.4. Comparison of the Radicle Inoculation and Inoculum Layer Methods

**Figure 3:**
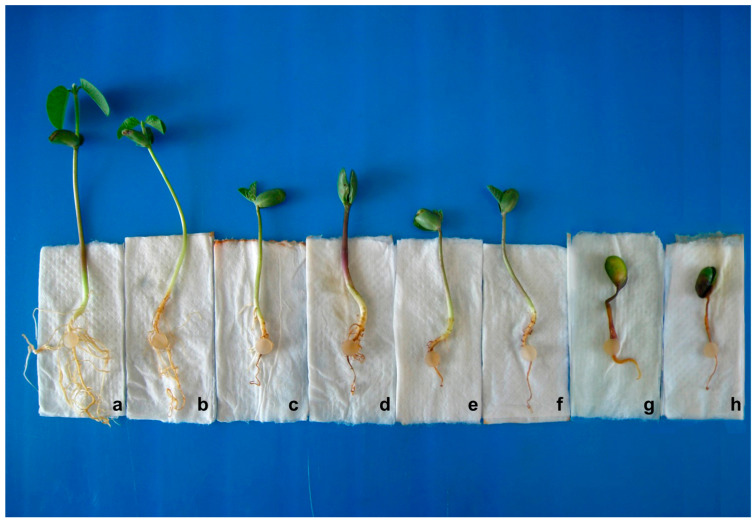
The reaction of roots after inoculation with *P. sojae* strain using radicle inoculation method. a—No visible disease symptoms on taproot and lateral roots (denoted as 0); b—Trace of rot on taproot and lateral roots (denoted as 1); c—Less than the bottom third of taproot mass rotted (denoted as 2); d—Bottom third of taproot mass rotted (denoted as 3); e—Bottom two-thirds of taproot mass rotted (denoted as 4); f—More than the bottom two-thirds of taproot mass rotted (denoted as 5); g—Taproot completely rotted with only a few lateral roots (denoted as 6); h—Taproot completely rotted without lateral roots + plant death (denoted as 7).

The radicle inoculation method was first used to evaluate the pathogenicity of *Fusarium graminearum* on soybean. It has several advantages, including being a simple time- and space-saving operation that produces stable results. In the present study, this technique was applied to identify partial resistance against *P. sojae*. We randomly selected race1, race13, and race54 for further identification of partial resistance by the radicle inoculation method. Ten days after inoculation, the roots of the plants were observed for different disease resistance reactions, some of which are shown in Figure 3. Similarly, the radicle inoculation method was graded on a scale of 0–7 and converted into a disease index for evaluating soybean cultivars for partial resistance. Soybean cultivars with consistent results for tolerance in both methods were counted, and their percentage of the total was calculated. The results showed that the consistency of the inoculum layer method and the radicle inoculation method for races 1, 13, and 54 were 96.00%, 84.38%, and 83.33%. Table S3 lists the disease index comparison of partial resistance evaluation by the inoculum layer method and radicle inoculation method. The above results suggest that the radicle inoculation method could potentially replace the inoculum layer method to identify partial resistance against *P. sojae*, and further verification with larger samples is required in the future.

## 3. Discussion

Since *P. sojae* was first discovered and isolated in Northeastern China in 1989 [21], many scholars have devoted themselves to the screening of germplasm resources for resistance to *P. sojae* and have confirmed the existence of abundant germplasm resources for resistance and concurrent resistance in China [27,28,29,30,31,32]. In the present study, 12 *P. sojae* strains were used to identify 112 short-season soybean cultivars from Northeast China for their resistance to PRR. Among them, 58 germplasms were resistant to 7–11 strains, accounting for 51.79% of the tested species. This result indicates abundant germplasm resources for disease resistance and multiple resistance in the Northeastern soybean production area and supports earlier reports from China [29,30,31,33]. Furthermore, it is noteworthy that only 23 germplasms showed resistance to the exotic strain USAR2. A possible explanation is that soybean resources from the same region may be similar in terms of genetic background and level of resistance [34]. Moreover, long-term coexistence and co-evolution of pathogens and hosts in the same place of origin could potentially result in fewer resistant resources in the absence of selection pressure. Therefore, using excellent resistant planting resources with different genetic backgrounds when cultivating disease-resistant cultivars can enrich the resistance diversity in Northeast China.

Among the tested materials in this study, 110 germplasms were resistant to 1–11 *P. sojae* strains, accounting for 98.21% of the total number of identifications. This information further shows that soybean cultivars commonly grown in Northeastern China are resistant to almost all *P. sojae* races. Previous studies have shown that *Rps1k*, *Rps1c,* and *Rps1a* have been widely used in breeding against *P. sojae* due to their stable broad-spectrum resistance, with *Rps1k* having the most stable and the highest broad-spectrum resistance [12,35,36,37]. Gene mining was performed by inoculating the hypocotyl to identify the results of resistance to susceptibility, producing a total of 92 types of anti-inductive responses. Mengdou 28 and Kejiao 10–262 may contain the *Rps1k*, *Rps1c*, and *Rps1a* genes. To the best of our knowledge, this is the first time that soybean germplasm containing the *Rps1k* gene has been derived from germplasm resources in Northeast China. Furthermore, 110 germplasms had unspecified genotypes, producing 91 reactivity types that were different from those containing a single known disease resistance gene or a combination of two known disease resistance genes. These cultivars may contain a new disease resistance gene or a combination of resistance genes. Using these cultivars in breeding programs will enable gene pyramiding in subsequent generations to develop multiple gene resistance for broader effectiveness against the pathogen.

Partial resistance is a highly heritable quantitative trait that is a valuable complement to major gene resistance. It limits the pathogen’s spot growth rate in the host tissue and reduces the severity of disease caused by *P. sojae*, thus limiting yield loss [19,20]. Further identification of partial resistance in the present study showed that the percentage of germplasm with high disease resistance accounted for 26.19–65.63% of the tested materials, confirming the presence of disease-resistant germplasm resources in Northeast China. However, partial resistance may not provide adequate control against a high number of pathogens [12]. The massive deployment of qualitative traits controlled by a single gene has resulted in higher selection pressure on the pathogen, thus shortening its available time. Therefore, incorporating *Rps* resistance into soybean genetic backgrounds with high levels of partial resistance may prolong the effective longevity of the *Rps* gene. Mining new disease resistance genes or combinations of disease resistance genes can provide excellent parents and vectors of superior genes for breeding cultivars resistant to PRR.

Since root resistance to *P. sojae* is a very important index in soybean breeding, the evaluation of root resistance is practical and valuable for the soybean industry. However, the research on soybean resistance to *P. sojae* is mainly focused on inoculating aboveground plant parts, usually hypocotyls, primarily for the inconvenience of investigating the roots in soil [38]. To date, there were only hydroponic inoculation procedure, aeroponics system, and inoculum layer test [38,39,40,41] reported to screen soybean root reaction to *P. sojae*, but all the above-mentioned methods were laborious and time-consuming when a large number of genotypes need to be evaluated against different *P. sojae* isolates. Therefore, an effective, fast, and reliable method to measure root resistance under controlled conditions would be very beneficial. In this study, the radicle inoculation method invented by Xue et al. [42] was also applied for the first time to screen soybean germplasm for partial resistance to *P. sojae*. We randomly selected race1, race13, and race54 for further identification of partial resistance by the radicle inoculation method and obtained more than 80% concordance. Benefits of the radicle inoculation method include better control over inoculation conditions, as well as over temperature and water levels for the disease, reduced infestation of foreign pests and diseases, and the possibility to record disease resistance in plants more accurately and conveniently. In addition, it can considerably reduce the amount of bacteria used during the procedure, effectively shorten the cycle of disease resistance, and save space. Therefore, this technique may become an alternative method for identifying disease resistance in future breeding programs; however, further validation with larger samples is still required.

## 4. Materials and Methods

### 4.1. Short-Season Soybean Cultivars

A total of 112 commercial soybean cultivars (lines) (Maturity Groups 000, 00, 0, and I; Table 2) were used to evaluate resistance to the 12 *P. sojae* races under greenhouse conditions. The breeding units that kindly provided these seeds are also listed in Table 2.

### 4.2. P. sojae Races

Isolates of 12 *P. sojae* races were used as inoculum for this work. *P. sojae* races 1, 3, 4, 5, 9, 13, 44, and 54 were isolated from soybean fields in Heilongjiang Province [28]. Races PsJs2, PsMC1, Ps41-1, and USAR2 were obtained from Dr. Zhendong Zhu (Institute of Crop Science, Chinese Academy of Agricultural Sciences). Throughout this investigation, isolates were kept on V8 juice agar plates at 25 °C for 7 days before being moved to fresh plates every 2 months. Additionally, all isolates were retested for virulence pre-inoculation. Table 3 contains a list of the virulence pathotypes against different cultivars.

### 4.3. Resistance Identification

Ten seeds of each cultivar (line) were grown in plastic pots (diameter = 10 cm) containing a soil: perlite:peat moss mixture (in a 1:1:1 volume ratio) in a greenhouse with a temperature range of 22–25 °C. Metal halide lamps of 300 W were used as additional lighting to maintain a 16 h photoperiod.

Seedlings at the first-node stage (V1) [35] were inoculated with *P. sojae* isolates using a hypocotyl wound technique described by Kaufmann and Gerdmann [5]. A blade was used to make a shallow cut along the hypocotyls of the seedlings, 1 cm below the cotyledon node. Next, the aerial mycelium was inserted into the longitudinal wound, which was taken from the edge of the pre-cultured *P. sojae* isolates.

Following inoculation, the plants were kept in a moist chamber for 3 days before being placed back in the growth chambers to monitor disease progression. Three replication pots with every ten plants were used in the experiment to gauge the response of soybean cultivars (lines) to different races. In order to test the adequacy of the environment for the development of infection and illness as well as the potential harm brought on by wounding plants, Williams (*rpsrps*) plants from three pots were wounded and inoculated with blank V8 juice agar without *P. sojae* isolates in each experiment. Five days after inoculation, the disease level of various soybean cultivars was examined. If a differential exhibited ≤30% seedling mortality, the reaction was considered resistance. If a differential exhibited ≥70% seedling mortality, the reaction was considered susceptible. Seedling mortality from 30% to 70% was considered an intermediate reaction [43].

### 4.4. Partial Resistance Reaction

Once a germplasm was identified as susceptible to *P. sojae* strains from the hypocotyl inoculation test, it was further evaluated for partial resistance to the strains using the inoculums layer method. Soybean plants were grown in plastic houses under natural conditions to ensure normal growth. Seeds of different soybean cultivars were inoculated with *P. sojae* isolates that involved placing the corresponding agar cultures underneath the seeds, according to the method described by Walker and Schmitthenner [44]. After three weeks, the degree of partial resistance of the different cultivars was assessed. The rating system for the layer test used a scale of 1 to 9, in which 1 = no root rot, 2 = a trace of root rot, 3 = the bottom third of root mass rotted, 4 = the bottom two-thirds of root mass rotted, 5 = all roots rooted + 10% of seedlings dead, 6 = 50% of seedlings dead + moderate stunting on tops, 7 = 75% of seedlings dead + severe stunting of tops, 8 = 90% of seedling dead, 9 = all seedlings dead. Referring to the identification criteria of Dorrance and Schmitthenner [45] and using the disease level formula of Liu [46] to transform into a disease index, it was concluded that a disease index ≤ 30 has high tolerance, a disease index from 30 to 60 has moderate tolerance, and a disease index ≥ 60 has a low tolerance.

### 4.5. Radicle Inoculation Techniques

The radicle inoculation techniques described by Xue et al. [42] for evaluating the pathogenicity of different *Fusarium* isolates on soybean seedlings were applied to evaluate the partial resistance of soybean to *P. sojae* in the present study. Each experiment’s seeds were sterilized by dipping them into 0.5% NaClO for 45 s and then giving them two rinses in sterile distilled water. After spreading the seeds evenly on two layers of sterile paper towels and moistening them with enough sterile water, the other two layers of sterile paper towels were laid on top to allow the seeds to germinate. When plants reached the early V1 growth stage [35] and root hairs became apparent, they were transferred to dark conditions at 25 °C and kept for 18 h. Then, visually sound seedlings were chosen and subjected to the same surface sterilization procedures as previously mentioned. Using a sterile metal needle, a 0.6 cm diameter agar plug was removed from the edge of the pre-cultured *P. sojae* isolates and inoculated approximately 1.5 cm behind the main root tip of the soybean seedling. Every inoculated plant was set on a pre-cut covering sheet, which was made of two layers of sterile tissue paper placed on a sheet of aluminum foil. The aluminum foil sheet was used to keep each unit separate and retain moisture. The trays were then placed in a growth chamber, and the water level in the tray was checked daily, and water was added as needed.

After 10 days of inoculation, the severity of root rot of different cultivars was assessed. The rating system employed a scale of 0–7, in which 0 = no visible disease symptoms on taproot and lateral roots, 1 = trace of rot on taproot and lateral roots, 2 = less than the bottom third of taproot mass rotted, 3 = bottom third of taproot mass rotted, 4 = bottom two-thirds of taproot mass rotted, 5 = more than the bottom two-thirds of taproot mass rotted, 6 = taproot completely rotted with only a few lateral roots, 7 = taproot completely rotted without lateral roots + plant death. Referring to the rating criteria of the inoculums layer method with slight modifications, the disease rating formula of Liu [46] was transformed into a disease index, and it was concluded that a disease index ≤20 has high tolerance, a disease index from 20 to 50 has moderate tolerance, and a disease index ≥50 has a low tolerance.

### 4.6. Statistical Analysis

Three replications of a randomized complete block design were used to arrange every pot. Residuals for each parameter in each experimental parameter were examined for normality and homogeneity of variances. The SAS UNIVARIATE technique was used to check the Shapiro–Wilk test’s assumption of normality, and the PLOT function was used to evaluate the random and homogeneous distribution of residuals (SAS Institute Inc., Cary, NC, USA, 2008).

## 5. Conclusions

In this study, we tested 112 short-season soybean cultivars from Northeast China for resistance to *P. sojae*. Combining the results of hypocotyl inoculation for resistance and the inoculum layer method for partial resistance, we screened a total of 35 superior soybean germplasm. Among these, Mengdou 28 and Kejiao 10–262 may harbor either *Rps3a* or multiple *Rps* genes conferring resistance to *P. sojae*. Furthermore, we used radicle inoculation for the first time to identify partial resistance to *P. sojae* and confirmed that the results were in high agreement with the inoculum layer method, which may serve as a time- and labor-saving method to identify partial resistance against *P. sojae* in the future. Our findings indicate that Northeast China is rich in excellent soybean germplasm resources resistant to *P. sojae* and provides a theoretical basis for screening candidate parental lines for PRR resistance breeding.

## Figures and Tables

**Table 1:** List of 35 candidate soybean cultivars that showed suitable resistance and partial resistance.

Cultivar (Line)	Number of *P. sojae* Resistance Strains	Disease Index	Cultivar (Line)	Number of *P. sojae* Resistance Strains	Disease Index
Suinong 35	11	19.71	Hefeng 52	7	15.92
Kejiao 10–262	11	26.33	Heihe 48	7	27.61
Mengdou 28	11	16.72	Mengdou 13	7	23.36
Dengke 4	11	13.58	Mengdou 32	7	20.28
Jiyu 35	10	23.26	Mengdou 33	7	23.04
Suinong 29	9	25.44	Mengdou 35	7	25.67
Dengke 1	9	29.85	Dengke 6	7	28.67
Suizhongzuo 40	8	24.60	Heinong 51	6	22.94
Beidou 42	8	27.68	Heihe 4	6	27.39
Heihe 29	8	29.85	Suinong 27	5	24.41
Mengdou 9	8	25.18	Suinong 38	5	25.41
Mengdou 11	8	18.88	Heihe 43	4	29.51
Mengdou 14	8	23.86	Kenfeng 16	4	28.99
Mengdou 37	8	28.33	Heihe 52	3	29.14
Mengdou 38	8	28.90	Heihe 35	2	26.72
Dengke 9	8	28.02	Mengdou 12	2	26.88
Dengke 10	8	19.85	Heinong 57	11	88.15
Hefeng 46	7	26.87			

**Table 2:** Source, maturity group, and heat unit of tested soybean genotypes.

Cultivar (Line)	Ins ^1^	HU ^2^	MG ^3^	Cultivar (Line)	Ins ^1^	HU ^2^	MG ^3^
Heinong 37	III	2600	I	Fengshou 27	XIV	2300	0
Heinong 46	III	2450	0	Dongnong 4400	VIII	2400	0
Heinong 51	III	2583	I	Dongnong 47	VIII	2400	0
Heinong 52	III	2550	I	Heihe 4	I	1950	00
Heinong 53	III	2600	I	Heihe 6	I	2100	00
Heinong 54	III	2400	0	Heihe 18	I	2150	00
Heinong 55	III	2600	I	Heihe 22	I	2050	00
Heinong 56	III	2380	0	Heihe 26	I	2100	00
Heinong 57	III	2500	0	Heihe 27	I	2100	00
Heinong 58	III	2400	0	Heihe 29	I	2000	00
Heinong 59	III	2400	0	Heihe 33	I	1900	000
Heinong 67	III	2400	0	Heihe 35	I	1780	000
Hefeng 35	II	2358	0	Heihe 36	I	2200	0
Hefeng 37	II	1885	000	Heihe 43	I	2150	00
Hefeng 39	II	2340	0	Heihe 45	I	2050	00
Hefeng 41	II	2427	0	Heihe 48	I	2180	00
Hefeng 44	II	2370	0	Heihe 50	I	2100	00
Hefeng 45	II	2347	0	Heihe 52	I	2150	00
Hefeng 46	II	2382	0	Heihe 53	I	2100	00
Hefeng 48	II	2281	0	Neidou 4	XIV	1900	000
Hefeng 50	II	2300	0	Mengdou 9	XIV	1950	00
Hefeng 51	II	2286	0	Mengdou 11	XIV	1900	000
Hefeng 52	II	2320	0	Mengdou 12	VI	1900	0
Hefeng 54	II	2320	0	Mengdou 13	VI	2300	0
Hefeng 55	II	2365	0	Mengdou 14	VI	2200	0
Suinong 14	IV	2450	0	Mengdou 15	VI	2200	0
Suinong 21	IV	2400	0	Mengdou 16	VI	2100	0
Suinong 22	IV	2400	0	Mengdou 26	VI	2300	0
Suinong 23	IV	2450	0	Mengdou 28	VI	2300	0
Suinong 24	IV	2280	0	Mengdou 30	VI	2400	0
Suinong 25	IV	2400	0	Mengdou 31	VI	2400	0
Suinong 26	IV	2400	0	Mengdou 32	VI	1900	000
Suinong 27	IV	2300	0	Mengdou 33	VI	2300	0
Suinong 28	IV	2400	0	Mengdou 34	VI	2000	00
Suinong 29	IV	2400	0	Mengdou 35	VI	2000	00
Suinong 31	IV	2400	0	Mengdou 36	VI	2200	0
Suinong 32	IV	2400	0	Mengdou 37	VI	1900	000
Suinong 33	IV	2400	0	Mengdou 38	VI	2100	0
Suinong 35	IV	2450	0	Dengke 1	VI	2100	0
Suinong 36	IV	2400	0	Dengke 3	VI	2100	0
Suinong 37	IV	2250	0	Dengke 4	VI	2300	0
Suinong 38	IV	2400	0	Dengke 5	XII	2100	0
Suizhongzuo 40	IV	2400	0	Dengke 6	XII	2000	00
Suinong 41	IV	2400	0	Dengke 9	XII	2200	0
Keshan 1	XIV	2100	0	Dengke 10	XII	2200	0
Kejiao 07–584	XIV	2400	0	Henong 60	IX	2500	0
Kejiao 08–952	XIV	2400	0	Henong 67	IX	2350	0
Kejiao 10–262	XIV	2350	0	Henong 75	IX	2400	0
Kejiao 10–2333	XIV	2400	0	Kenfeng 16	VII	2447	0
Kejiao 10–2192	XIV	2350	0	Kendou 6	VII	2250	0
Beidou 9	XIII	2300	0	Kenjiandou 28	VII	2260	0
Beidou 42	V	2100	0	Kenfeng 22	VII	2250	0
Beidou 48	V	2250	0	Kennong 5	VII	2368	0
Beifeng 11	XIII	2208	0	Jiyu 35	X	2500	I
Fengshou 23	III	1800	000	Jiyu 97	X	2550	I
Fengshou 24	XIV	2200	0	Hongfeng 3	XI	2100	00

Note: ^1^ Seed breeding unit (I: Heihe Branch, Heilongjiang Academy of Agricultural Sciences; II: Heilongjiang Agricultural Institute, Heilongjiang Academy of Agricultural Sciences; III: Heilongjiang Academy of Agricultural Sciences; IV: Suihua Institute of Agricultural Sciences, Heilongjiang Academy of Agricultural Sciences; V: Huajiang Institute, The Crop Research and Breeding Center of Land Reclamation of Heilongjiang Province; VI: Hunlunbeier Institute of Agricultural Sciences; VII: Crop Research Institute, Land-Reclaimable Sciences of Heilongjiang Province; VIII: Soybean Research Institute, Northeast Agricultural University; IX: Jiamusi Branch of the Heilongjiang Academy of Agricultural Sciences; X: Jilin Academy of Agricultural Sciences; XI: HongXingLong Farm; XII: Mo Qi Dengke Seed Industry LLC; XIII: Beian Institute of Agricultural Sciences, Land Reclamation of Heilongjiang; XIV: Keshan Branch of the Heilongjiang Academy of Agricultural Sciences); ^2^ Heat Unit; ^3^ Maturity Group.

**Table 3:** Virulence reaction of *P. sojae* strains on differential hosts.

Differential Host	Resistance Gene	*P. sojae* Stains											
		1	3	4	5	9	13	44	54	PsJS2	PsMC1	Ps41-1	USAR2
L75-6141	*Rps*1a	R	S	S	S	S	R	S	R	S	S	S	R
L77-1863	*Rps*1b	R	R	R	R	R	R	R	R	S	R	R	S
L75-3735	*Rps*1c	R	R	S	S	R	R	R	R	S	S	R	R
P.I.103	*Rps*1d	R	R	R	R	R	R	S	S	S	R	S	R
Williams 82	*Rps*1k	R	R	R	R	R	R	R	R	S	S	R	R
L83-70	*Rps*3a	R	R	R	R	R	R	R	R	S	R	R	R
L89-1581	*Rps*6	R	R	R	S	S	S	R	R	S	S	R	R
L93-3258	*Rps*7	S	S	S	S	S	S	S	S	S	S	S	S
Williams	*rps*	S	S	S	S	S	S	S	S	S	S	S	S
Pathotype		7	1a, 7	1a, 1c, 7	1a, 1c, 6, 7	1a, 6, 7	6, 7	1a, 1d, 7	1d, 7	1a, 1b, 1c, 1d, 1k, 3a, 3b, 3c, 4, 5, 6, 7, 8	1a, 1c, 1k, 2, 3b, 3c, 4, 5, 6, 7, 8	1a, 1d, 2, 3b, 3c, 5, 7, 8	1b, 2, 3c, 5, 7

Note: R = resistant, S = susceptible.

## Data Availability

All data are represented in the article’s Supplementary tables.

## Supplementary Materials

The following supporting information can be downloaded at: https://www.mdpi.com/article/10.3390/ijms24076027/s1.
