# Supplementary material for: Evaluation of Short-Season Soybean Genotypes for Resistance and Partial Resistance to Phytophthora sojae
Source: Int J Mol Sci. 2023 Mar 23;24(7):6027. doi: 10.3390/ijms24076027 (PMC10093987; doi:10.3390/ijms24076027)
Supplement: Supplementary file 1 [file ijms-24-06027-s001.zip › ijms-2133736-supplementary.pdf]

Table S1. Reaction pattern of 112 soybean cultivars to 12 *P. sojae* strains

Germplasm	Reaction pattern	Resistance gene (*RPS*)
Mengdou 28, Kejiao 10-262	$R_1R_2R_3R_4R_5R_6R_7R_8S_9R_{10}R_{11}R_{12}$	3a, 1a+3a, 1a+1b, 1b+1c, 1b+1d, 1b+3a, 1b+6, 1c+1d, 1c+3a, 1d+1k, 1d+3a, 1d+6
Heinong 53	$S_1S_2S_3S_4S_5R_6S_7S_8S_9S_{10}S_{11}S_{12}$	
Suinong 28, Henong 60	$S_1S_2S_3S_4S_5S_6S_7S_8S_9S_{10}S_{11}S_{12}$	
Kendou 6, Dongnong 4400	$S_1S_2S_3S_4S_5S_6S_7S_8R_9R_{10}S_{11}S_{12}$	
Heihe 35	$S_1S_2S_3S_4S_5R_6S_7R_8S_9S_{10}S_{11}S_{12}$	
Heinong 46	$S_1S_2S_3S_4S_5R_6S_7R_8R_9R_{10}S_{11}R_{12}$	
Heinong 56	$S_1S_2S_3S_4S_5R_6S_7R_8R_9R_{10}S_{11}S_{12}$	
Jiyu 97	$S_1S_2S_3S_4S_5R_6S_7R_8S_9R_{10}S_{11}S_{12}$	
Heinong 58	$S_1S_2S_3S_4S_5R_6R_7R_8R_9R_{10}S_{11}S_{12}$	
Hefeng 37, Hefeng 51	$S_1S_2S_3S_4S_5S_6S_7S_8R_9S_{10}S_{11}S_{12}$	
Suinong 23	$S_1S_2S_3S_4S_5S_6R_7R_8S_9R_{10}R_{11}R_{12}$	
Heihe 52	$S_1S_2S_3S_4R_5S_6S_7S_8R_9R_{10}S_{11}S_{12}$	
Kenfeng 16	$S_1S_2S_3S_4R_5R_6S_7R_8R_9S_{10}S_{11}S_{12}$	
Hefeng 45	$S_1S_2R_3S_4S_5R_6R_7S_8S_9S_{10}S_{11}S_{12}$	
Hefeng 55	$S_1S_2R_3S_4S_5S_6S_7R_8S_9S_{10}S_{11}S_{12}$	
Heinong 37	$R_1S_2S_3R_4S_5R_6S_7R_8S_9R_{10}S_{11}S_{12}$	
Hefeng 52	$R_1S_2R_3R_4R_5S_6R_7R_8R_9S_{10}S_{11}S_{12}$	
Heihe 27	$S_1S_2R_3R_4S_5S_6S_7R_8R_9R_{10}S_{11}S_{12}$	
Mengdou 12	$S_1S_2R_3S_4R_5S_6S_7S_8S_9S_{10}S_{11}S_{12}$	
Hefeng 54	$S_1R_2S_3S_4R_5S_6S_7S_8S_9S_{10}S_{11}S_{12}$	
Mengdou 31	$S_1R_2R_3S_4R_5S_6S_7S_8S_9S_{10}S_{11}S_{12}$	
Suinong 25	$S_1R_2S_3S_4R_5S_6S_7R_8S_9R_{10}S_{11}S_{12}$	
Beifeng 11	$S_1R_2S_3R_4R_5R_6R_7R_8S_9R_{10}R_{11}S_{12}$	
Beidou 42	$S_1R_2R_3S_4R_5R_6S_7R_8R_9S_{10}R_{11}R_{12}$	
Suinong 38	$R_1S_2S_3S_4R_5S_6S_7R_8R_9R_{10}S_{11}S_{12}$	
Heinong 59	$R_1S_2S_3S_4R_5S_6S_7R_8S_9R_{10}S_{11}S_{12}$	
Heinong 52	$R_1S_2S_3S_4S_5S_6S_7S_8R_9R_{10}S_{11}S_{12}$	
Suinong 14	$R_1S_2S_3S_4S_5R_6S_7R_8S_9R_{10}S_{11}S_{12}$	
Suinong 31, Kenjiandou 28	$R_1S_2S_3S_4R_5R_6S_7R_8R_9S_{10}S_{11}S_{12}$	
Heihe 36	$R_1S_2S_3S_4R_5R_6S_7R_8S_9R_{10}S_{11}S_{12}$	
Heihe 53	$R_1S_2S_3S_4R_5R_6S_7R_8R_9R_{10}S_{11}S_{12}$	
Heihe 43, Heinong 54	$R_1S_2S_3S_4R_5R_6S_7R_8S_9S_{10}S_{11}S_{12}$	
Suinong 37	$R_1S_2S_3S_4R_5R_6R_7R_8R_9S_{10}S_{11}S_{12}$	
Heihe 4	$R_1S_2S_3S_4R_5S_6R_7R_8S_9R_{10}S_{11}R_{12}$	
Dengke 1	$R_1S_2R_3R_4R_5R_6R_7R_8R_9R_{10}S_{11}S_{12}$	
Kejiao 10-2192	$R_1S_2S_3R_4R_5R_6R_7R_8S_9R_{10}S_{11}S_{12}$	
Heinong 51	$R_1S_2S_3R_4R_5R_6S_7R_8R_9S_{10}S_{11}S_{12}$	
Dengke 4	$R_1R_2R_3R_4R_5S_6R_7R_8R_9R_{10}R_{11}R_{12}$	
Hefeng 35	$R_1S_2S_3R_4R_5R_6S_7S_8S_9S_{10}S_{11}S_{12}$	

Table S1. (Continued from previous page)

Germplasm	Reaction pattern	Resistance gene (*RPS*)
Hongfeng 3	$R_1S_2S_3R_4R_5S_6S_7R_8S_9S_{10}S_{11}R_{12}$	
Suinong 24	$R_1S_2R_3S_4R_5S_6S_7S_8S_9S_{10}S_{11}S_{12}$	
Dengke 10	$R_1S_2S_3R_4R_5R_6S_7S_8R_9R_{10}R_{11}R_{12}$	
Heihe 22, Kenfeng 22	$R_1S_2R_3S_4R_5R_6S_7R_8S_9S_{10}S_{11}S_{12}$	
Mengdou 14	$R_1S_2R_3S_4R_5R_6R_7R_8S_9R_{10}R_{11}S_{12}$	
Suinong 41	$R_1S_2R_3S_4R_5R_6R_7R_8R_9R_{10}S_{11}S_{12}$	
Suinong 29	$R_1R_2S_3R_4R_5S_6S_7R_8R_9R_{10}R_{11}R_{12}$	
Suinong 35	$R_1R_2S_3R_4R_5R_6R_7R_8R_9R_{10}R_{11}R_{12}$	
Mengdou 33	$R_1S_2R_3S_4R_5R_6R_7R_8S_9R_{10}S_{11}S_{12}$	
Heihe 29	$R_1S_2R_3S_4R_5R_6R_7R_8R_9S_{10}S_{11}R_{12}$	
Hefeng 44	$R_1S_2R_3R_4R_5R_6S_7S_8S_9S_{10}S_{11}S_{12}$	
Fengshou 27	$R_1S_2R_3R_4R_5R_6S_7R_8S_9S_{10}R_{11}R_{12}$	
Heihe 33	$R_1S_2R_3R_4R_5R_6S_7R_8R_9S_{10}S_{11}R_{12}$	
Heihe 26	$R_1S_2R_3R_4R_5R_6S_7R_8S_9R_{10}S_{11}S_{12}$	
Heinong 67	$R_1S_2R_3R_4R_5R_6S_7R_8S_9S_{10}S_{11}S_{12}$	
Mengdou 15	$R_1S_2R_3R_4R_5S_6R_7R_8S_9R_{10}R_{11}R_{12}$	
Mengdou 38, Mengdou 36	$R_1S_2R_3R_4R_5R_6R_7R_8S_9R_{10}S_{11}S_{12}$	
Mengdou 13, Mengdou 16, Mengdou 30, Mengdou 34, Mengdou 35 and Mengdou 26	$R_1S_2R_3R_4R_5R_6R_7R_8S_9S_{10}S_{11}S_{12}$	
Dengke 3, Kejiao 07-584	$R_1S_2R_3R_4R_5R_6R_7R_8S_9R_{10}R_{11}R_{12}$	
Mengdou 9	$R_1S_2R_3R_4R_5R_6R_7R_8R_9S_{10}S_{11}S_{12}$	
Suinong 22	$R_1R_2S_3S_4R_5R_6S_7R_8S_9R_{10}R_{11}R_{12}$	
Suizhongzuo 40	$R_1R_2S_3S_4R_5R_6S_7R_8R_9R_{10}R_{11}S_{12}$	
Dongnong 47	$R_1R_2S_3S_4R_5R_6S_7R_8S_9S_{10}S_{11}S_{12}$	
Hefeng 46	$R_1R_2S_3S_4R_5R_6S_7R_8R_9R_{10}S_{11}S_{12}$	
Suinong 27	$R_1R_2S_3S_4R_5S_6S_7S_8S_9R_{10}S_{11}R_{12}$	
Fengshou 24	$R_1R_2S_3S_4R_5S_6S_7R_8R_9S_{10}S_{11}S_{12}$	
Heihe 45	$R_1R_2S_3S_4R_5S_6S_7R_8S_9S_{10}S_{11}S_{12}$	
Mengdou 32, Hefeng 41	$R_1R_2S_3R_4S_5R_6S_7R_8S_9R_{10}S_{11}R_{12}$	
Heihe 6	$R_1R_2S_3S_4R_5S_6S_7R_8R_9R_{10}S_{11}R_{12}$	
Beidou 9	$S_1S_2R_3R_4S_5R_6R_7R_8S_9S_{10}S_{11}S_{12}$	
Dengke 5	$R_1R_2S_3R_4R_5S_6S_7S_8S_9S_{10}R_{11}S_{12}$	
Heinong 55	$R_1R_2S_3R_4S_5R_6S_7S_8S_9S_{10}R_{11}R_{12}$	
Hefeng 50	$R_1R_2S_3R_4R_5R_6S_7R_8S_9R_{10}S_{11}S_{12}$	
Heihe 18	$R_1R_2S_3R_4R_5R_6S_7R_8S_9S_{10}S_{11}S_{12}$	
Henong 67, Suinong 33	$R_1R_2S_3R_4R_5R_6S_7R_8S_9R_{10}R_{11}R_{12}$	
Hefeng 48	$R_1R_2S_3R_4R_5S_6S_7S_8R_9R_{10}R_{11}R_{12}$	
Neidou 4	$R_1R_2S_3R_4R_5R_6R_7R_8S_9R_{10}S_{11}R_{12}$	
Suinong 36	$R_1R_2S_3R_4R_5R_6R_7R_8R_9R_{10}S_{11}S_{12}$	
Kennong 5	$R_1R_2R_3S_4S_5R_6R_7R_8S_9S_{10}S_{11}S_{12}$	

Table S1. (Continued from previous page)

Germplasm	Reaction pattern	Resistance gene (*RPS*)
Kejiao 08-952	$R_1R_2R_3S_4R_5R_6S_7R_8S_9R_{10}R_{11}S_{12}$	
Heinong 57	$R_1R_2R_3R_4S_5R_6R_7R_8R_9R_{10}R_{11}R_{12}$	
Hefeng 39	$R_1R_2R_3R_4R_5S_6S_7R_8S_9R_{10}R_{11}R_{12}$	
Dengke 9	$R_1R_2R_3R_4S_5R_6R_7R_8S_9R_{10}S_{11}S_{12}$	
Suinong 21	$R_1R_2S_3R_4S_5S_6S_7S_8S_9R_{10}R_{11}R_{12}$	
Heihe 48, Dengke 6	$R_1R_2R_3R_4R_5R_6S_7R_8S_9S_{10}S_{11}S_{12}$	
Fengshou 23	$R_1R_2R_3R_4R_5R_6S_7R_8R_9R_{10}S_{11}S_{12}$	
Suinong 32, Henong 75	$R_1R_2R_3R_4R_5R_6S_7R_8S_9R_{10}R_{11}R_{12}$	
Mengdou 37, Kejiao 10-2333 and Mengdou 11	$R_1R_2R_3R_4R_5R_6S_7R_8S_9S_{10}S_{11}R_{12}$	
Jiyu 35	$R_1R_2R_3R_4S_5R_6S_7R_8R_9R_{10}R_{11}R_{12}$	
Heihe 50	$R_1R_2R_3R_4R_5R_6R_7S_8S_9S_{10}S_{11}S_{12}$	
Keshan 1	$R_1R_2R_3R_4R_5R_6R_7R_8S_9S_{10}S_{11}S_{12}$	
Suinong 26	$R_1R_2S_3R_4S_5R_6S_7S_8S_9R_{10}S_{11}S_{12}$	
Beidou 48	$R_1R_2R_3R_4R_5R_6R_7R_8R_9R_{10}S_{11}S_{12}$	

Note: [1]race 1, [2]race 3, [3]race 4, [4]race 5, [5]race 9, [6]race 13, [7]race 44, [8]race 54, [9]PsJS2, [10]PsMC1, [11]Ps41-1, [12]USAR2.

Table S2. Evaluation of soybean germplasm with partial resistance to *P. sojae* strains using inoculums layer method

Germplasm	Disease index (DI)[Z]												
	race1	race3	race4	race5	race9	race13	race44	race54	PsJs2	PsMC1	Ps41-1	USAR2	Mean
Heinong 37	—	83.33	82.35	—	43.83	—	81.75	—	30.50	—	58.73	29.63	58.59
Heinong 46	75.06	79.63	37.49	83.70	72.59	—	82.69	—	—	—	86.30	—	73.92
Heinong 51	—	13.89	12.40	—	—	—	22.93	—	—	33.28	11.11	44.03	22.94
Heinong 52	—	52.78	45.37	50.21	81.75	58.99	19.29	68.75	—	—	46.34	41.37	51.65
Heinong 53	41.73	28.35	87.41	78.85	78.85	—	29.05	45.37	45.74	17.77	38.58	38.68	48.22
Heinong 54	—	47.22	36.16	33.33	—	—	58.99	—	46.33	51.56	63.36	30.26	45.90
Heinong 55	—	—	40.37	—	19.29	—	81.75	39.63	38.43	28.43	—	—	41.32
Heinong 56	43.62	34.04	42.12	44.85	34.39	—	19.29	—	—	—	49.38	24.21	36.49
Heinong 57	—	—	—	—	88.15	—	—	—	—	—	—	—	88.15

Table S2. (Continued from previous page)

Germplasm	Disease index (DI)[Z]												
	race1	race3	race4	race5	race9	race13	race44	race54	PsJs2	PsMC1	Ps41-1	USAR2	Mean
Heinong 58	51.11	53.48	52.22	53.33	82.22	—	—	—	—	—	78.61	67.90	62.70
Heinong 59	—	58.36	48.70	46.11	—	80.22	68.44	—	45.30	—	53.70	40.17	55.13
Heinong 67	—	23.46	—	—	—	—	30.29	—	68.77	47.89	46.48	11.11	38.00
Hefeng 35	—	16.67	12.91	—	—	—	48.26	13.40	61.46	86.90	32.10	16.72	36.05
Hefeng 37	47.53	43.09	71.97	86.90	30.09	33.33	43.52	71.60	—	18.52	38.52	56.94	49.27
Hefeng 39	—	—	—	—	—	70.16	54.32	—	61.47	—	—	—	61.98
Hefeng 41	—	—	41.73	—	58.99	—	57.87	—	41.59	—	29.28	—	45.89
Hefeng 44	—	25.51	—	—	—	—	81.75	59.54	36.42	27.88	59.72	22.22	44.72
Hefeng 45	40.95	14.91	—	55.56	50.95	—	—	28.52	50.40	37.65	69.14	45.91	43.78
Hefeng 46	—	—	16.42	39.11	—	—	13.42	—	—	—	26.79	38.62	26.87
Hefeng 48	—	—	82.55	—	—	29.26	31.75	41.93	—	—	—	—	46.37
Hefeng 50	—	—	36.63	—	—	—	69.18	—	36.67	—	42.96	27.78	42.64
Hefeng 51	38.78	26.30	67.42	62.72	62.24	31.85	35.24	15.93	—	20.99	52.10	41.21	41.34
Hefeng 52	—	19.38	—	—	—	13.83	—	—	—	17.18	12.35	16.87	15.92
Hefeng 54	23.05	—	47.22	48.77	—	19.29	55.40	19.29	26.85	43.52	37.57	37.37	35.83
Hefeng 55	41.72	25.19	—	84.44	62.47	21.77	62.22	—	24.07	21.10	37.04	14.44	39.45
Suinong 14	—	30.68	34.52	55.09	13.23	—	25.68	—	17.92	—	24.07	66.01	33.40
Suinong 21	—	—	21.11	—	78.89	13.33	38.15	14.81	67.48	—	—	—	38.96
Suinong 22	—	—	32.61	32.06	—	—	59.54	—	43.27	—	—	—	41.87
Suinong 23	20.16	18.31	46.50	22.22	59.54	74.73	—	—	42.80	—	—	—	40.61

Table S2. (Continued from previous page)

Germplasm	Disease index (DI)^{Z}												
	race1	race3	race4	race5	race9	race13	race44	race54	PsJs2	PsMC1	Ps41-1	USAR2	Mean
Suinong 24	—	33.37	—	65.15	—	22.55	21.73	36.63	74.92	24.07	51.09	30.74	40.03
Suinong 25	11.11	—	41.73	22.22	—	71.06	57.87	—	41.59	—	29.81	23.05	37.31
Suinong 26	—	—	14.89	—	17.28	—	83.70	40.10	48.40	—	24.34	13.82	34.65
Suinong 27	—	—	15.24	11.11	—	16.05	22.91	27.04	17.07	—	61.48	—	24.41
Suinong 28	16.67	16.93	16.91	52.72	11.11	29.45	12.26	15.94	41.41	30.97	79.37	41.98	30.48
Suinong 29	—	—	18.91	—	—	31.11	26.30	—	—	—	—	—	25.44
Suinong 31	—	22.63	52.98	42.22	—	—	26.67	—	—	24.81	67.90	12.96	35.74
Suinong 32	—	—	—	—	—	—	70.99	—	42.96	—	—	—	56.98
Suinong 33	—	—	79.01	—	—	—	28.77	—	23.99	—	—	—	43.92
Suinong 35	—	—	19.71	—	—	—	—	—	—	—	—	—	19.71
Suinong 36	—	—	38.12	—	—	—	—	—	—	—	61.19	32.51	43.94
Suinong 37	—	24.39	80.32	46.60	—	—	—	—	—	18.83	77.41	18.64	44.37
Suinong 38	—	15.72	21.44	16.75	—	24.54	22.22	—	—	—	60.13	17.06	25.41
Suizhongzuo 40	—	—	36.78	19.64	—	—	27.78	—	—	—	—	14.20	24.60
Suinong 41	—	22.55	—	16.87	—	—	—	—	—	—	76.79	11.93	32.04
Keshan 1	—	—	—	—	—	—	—	—	11.93	21.56	25.93	47.90	31.04
Kejiao 07-584	—	50.18	—	—	—	—	—	—	53.43	—	—	—	51.81
Kejiao 08-952	—	—	—	11.11	—	—	61.47	—	42.39	—	—	41.45	39.11
Kejiao 10-2192	—	38.13	11.11	—	—	—	—	—	26.35	—	56.54	45.93	35.61
Kejiao 10-2333	—	—	—	—	—	—	70.16	—	37.64	11.11	59.26	—	44.54

Table S2. (Continued from previous page)

Germplasm	Disease index (DI)[Z]												
	race1	race3	race4	race5	race9	race13	race44	race54	PsJs2	PsMC1	Ps41-1	USAR2	Mean
Kejiao 10-262	—	—	—	—	—	—	—	—	26.33	—	—	—	26.33
Beidou 9	19.54	18.52	—	—	11.11	—	—	—	39.88	46.43	43.62	41.67	31.54
Beidou 42	11.11	—	—	11.11	—	—	37.10	—	—	51.39	—	—	27.68
Beidou 48	—	—	—	—	—	—	—	—	—	—	66.85	34.92	50.89
Beifeng 11	17.06	—	11.11	—	—	—	—	—	76.19	—	—	46.60	37.74
Fengshou 23	—	—	—	—	—	—	13.40	—	—	—	47.89	40.49	33.93
Fengshou 24	—	—	29.63	22.22	—	73.54	16.54	—	—	37.10	49.38	11.11	34.22
Fengshou 27	—	14.79	—	—	—	—	13.40	—	40.12	70.16	—	—	34.62
Dongnong 4400	21.07	14.92	13.84	71.09	13.27	63.46	54.07	15.48	—	—	56.79	39.47	36.35
Heihe 4	—	12.41	14.57	12.96	—	24.75	—	—	44.53	—	55.11	—	27.39
Heihe 6	—	—	33.80	30.48	—	71.43	20.41	—	—	—	14.51	—	34.13
Dongnong 47	—	—	13.39	77.78	—	—	23.33	—	19.26	76.95	18.40	38.44	38.22
Heihe 18	—	—	12.70	—	—	—	56.91	—	12.04	29.20	74.63	32.10	36.26
Heihe 22	—	16.67	—	36.42	—	—	48.26	—	61.46	86.90	32.10	16.72	42.65
Heihe 26	—	17.28	—	—	—	—	53.92	—	65.28	—	55.97	50.85	48.66
Heihe 27	55.56	39.86	—	—	30.30	37.57	37.10	—	—	—	65.61	11.85	39.69
Heihe 29	—	13.24	—	28.89	—	—	—	—	—	34.81	42.46	—	29.85
Heihe 33	—	21.14	—	—	—	—	13.42	—	—	61.47	26.79	—	30.71
Heihe 35	38.07	12.41	14.57	12.96	12.35	—	34.39	—	44.53	28.50	55.11	14.35	26.72
Heihe 36	—	21.14	16.42	43.83	—	—	13.42	—	70.16	—	26.79	38.62	32.91

Table S2. (Continued from previous page)

Germplasm	Disease index (DI)^{Z}												
	race1	race3	race4	race5	race9	race13	race44	race54	PsJs2	PsMC1	Ps41-1	USAR2	Mean
Heihe 43	—	24.26	22.10	34.60	—	—	39.54	—	41.98	18.88	19.07	35.65	29.51
Heihe 45	—	—	63.81	54.91	—	35.31	55.09	—	49.17	15.66	79.20	12.70	45.73
Heihe 48	—	—	—	—	—	—	27.18	—	27.74	16.14	44.75	22.22	27.61
Heihe 50	—	—	—	—	—	—	—	37.04	70.90	70.16	62.88	11.11	50.42
Heihe 52	38.14	23.10	20.78	55.40	—	51.85	24.41	11.11	—	—	17.39	20.06	29.14
Heihe 53	—	31.57	51.59	48.61	—	—	40.93	—	—	—	55.09	18.52	41.05
Neidou 4	—	—	13.76	—	—	—	—	—	15.59	—	52.34	—	33.05
Mengdou 9	—	13.89	—	—	—	—	—	—	—	31.69	11.11	44.03	25.18
Mengdou 11	—	—	—	—	—	—	22.91	—	17.07	16.67	61.48	—	18.88
Mengdou 12	24.24	23.05	—	18.27	—	17.05	65.07	20.97	14.71	22.09	17.20	46.16	26.88
Mengdou 13	—	45.47	—	—	—	—	—	—	29.73	19.36	11.11	11.11	23.36
Mengdou 14	—	20.33	—	36.73	—	—	—	—	24.94	—	—	13.43	23.86
Mengdou 15	—	19.91	—	—	—	65.61	—	—	37.10	—	—	—	40.87
Mengdou 16	—	39.51	—	—	—	—	—	—	54.06	59.01	61.47	25.99	48.01
Mengdou 26	—	11.11	—	—	—	—	—	—	78.71	38.89	11.93	31.57	34.44
Mengdou 28	—	—	—	—	—	—	—	—	16.72	—	—	—	16.72
Mengdou 30	—	38.35	—	—	—	—	—	—	56.48	57.30	54.94	44.81	50.38
Mengdou 31	21.69	—	—	59.55	—	51.65	35.65	40.14	54.50	37.59	61.47	33.13	43.93
Mengdou 32	—	—	11.85	—	16.84	—	11.85	—	17.49	—	43.38	—	20.28
Mengdou 33	—	15.28	—	16.20	—	—	—	—	14.66	—	34.07	35.01	23.04

Table S2. (Continued from previous page)

Germplasm	Disease index (DI)^{Z}												
	race1	race3	race4	race5	race9	race13	race44	race54	PsJs2	PsMC1	Ps41-1	USAR2	Mean
Mengdou 34	—	42.06	—	—	—	—	—	—	16.72	53.54	54.94	31.48	39.75
Mengdou 35	—	17.16	—	—	—	—	—	—	30.26	31.79	26.19	22.93	25.67
Mengdou 36	—	26.70	—	—	—	—	—	—	61.47	—	16.72	18.52	30.85
Mengdou 37	—	—	—	—	—	—	15.56	—	25.39	28.57	43.80	—	28.33
Mengdou 38	—	18.52	—	—	—	—	—	—	61.01	—	24.94	11.11	28.90
Dengke 1	—	37.04	—	—	—	—	—	—	—	—	37.10	15.41	29.85
Dengke 3	—	11.11	—	—	—	—	—	—	54.94	—	—	—	33.03
Dengke 4	—	—	—	—	—	13.58	—	—	—	—	—	—	13.58
Dengke 5	—	—	42.86	—	—	61.06	34.20	49.85	34.17	45.30	—	31.90	42.76
Dengke 6	—	—	—	—	—	—	20.99	—	19.71	30.55	59.16	12.96	28.67
Dengke 9	—	—	—	—	11.11	—	—	—	32.61	—	33.98	34.36	28.02
Dengke 10	—	23.10	20.78	—	—	—	24.41	11.11	—	—	—	—	19.85
Henong 60	42.22	40.00	41.30	39.51	60.00	25.56	69.49	86.90	79.38	49.55	78.89	61.23	56.17
Henong 67	—	—	46.30	—	—	—	26.76	—	23.23	—	—	—	32.10
Henong 75	—	—	—	—	—	—	27.88	—	50.58	—	—	—	39.23
Kenfeng 16	34.81	19.63	38.93	24.53	—	—	31.67	—	—	17.04	52.37	12.96	28.99
Kendou 6	42.59	20.37	57.05	17.70	13.24	24.60	29.75	26.91	—	—	60.82	14.63	30.77
Kenjiandou 28	—	44.44	15.19	17.72	—	—	59.94	—	—	45.93	86.73	29.63	42.80
Kenfeng 22	—	25.68	—	41.45	—	—	38.07	—	78.80	24.94	29.17	11.11	35.60
Kennong 5	—	—	—	11.11	56.91	—	—	—	35.21	29.63	41.42	11.11	30.90
Jiyu 35	—	—	—	—	30.00	—	16.52	—	—	—	—	—	23.26

Table S2. (Continued from previous page)

Germplasm	Disease index (DI)^{Z}												
	race1	race3	race4	race5	race9	race13	race44	race54	PsJs2	PsMC1	Ps41-1	USAR2	Mean
Jiyu 97	21.23	72.35	84.10	81.75	56.30	—	37.04	—	32.04	—	61.30	20.52	51.85
Hongfeng 3	—	13.22	50.91	—	—	12.96	24.85	—	31.16	38.02	39.14	—	30.04

Note：^{Z} Values are the average of three experiments. '—' =Once a germplasm was identified as resistance to *P. sojae* strains in hypocotyl inoculation test, the germplasm was not evaluated for partial resistance. The grey part of the table shows soybean cultivars with high partial resistance.

Table S3. Comparison of partial resistance evaluation of randomly selected races inoculated by inoculum layer method and radicle inoculation method

Germplasm	Disease index (DI)^{Z}					
	race1		race13		race54	
	ILM	RIM	ILM	RIM	ILM	RIM
Heinong 37	—	—	—	—	—	—
Heinong 46	75.06	68.15	—	—	—	—
Heinong 51	—	—	—	—	—	—
Heinong 52	—	—	58.99	49.63	68.75	57.78
Heinong 53	41.73	28.89	—	—	45.37	20.00
Heinong 54	—	—	—	—	—	—
Heinong 55	—	—	—	—	39.63	30.37
Heinong 56	43.62	32.59	—	—	—	—
Heinong 57	—	—	—	—	—	—
Heinong 58	51.11	45.19	—	—	—	—
Heinong 59	—	—	80.22	68.89	—	—
Heinong 67	—	—	—	—	—	—
Hefeng 35	—	—	—	—	13.40	1.48
Hefeng 37	47.53	33.33	33.33	24.45	71.60	69.63
Hefeng 39	—	—	70.16	59.26	—	—
Hefeng 41	—	—	—	—	—	—
Hefeng 44	—	—	—	—	59.54	45.19
Hefeng 45	40.95	25.19	—	—	28.52	14.08
Hefeng 46	—	—	—	—	—	—
Hefeng 48	—	—	29.26	14.82	41.93	19.26
Hefeng 50	—	—	—	—	—	—
Hefeng 51	38.78	22.22	31.85	17.04	15.93	11.11
Hefeng 52	—	—	13.83	1.48	—	—

Table S3. (Continued from previous page)

Germplasm	Disease index (DI)^{Z}					
	race1		race13		race54	
	ILM	RIM	ILM	RIM	ILM	RIM
Hefeng 54	23.05	11.11	19.29	7.41	19.29	6.67
Hefeng 55	41.72	27.42	21.77	12.59	—	—
Suinong 14	—	—	—	—	—	—
Suinong 21	—	—	13.33	0.74	14.81	11.11
Suinong 22	—	—	—	—	—	—
Suinong 23	20.16	11.11	74.73	62.96	—	—
Suinong 24	—	—	22.55	9.63	36.63	15.56
Suinong 25	11.11	0.00	71.06	57.04	—	—
Suinong 26	—	—	—	—	40.10	27.41
Suinong 27	—	—	16.05	6.67	27.04	15.56
Suinong 28	16.67	9.63	29.45	3.70	15.94	34.82
Suinong 29	—	—	31.11	20.00	—	—
Suinong 31	—	—	—	—	—	—
Suinong 32	—	—	—	—	—	—
Suinong 33	—	—	—	—	—	—
Suinong 35	—	—	—	—	—	—
Suinong 36	—	—	—	—	—	—
Suinong 37	—	—	—	—	—	—
Suinong 38	—	—	24.54	17.04	—	—
Suizhongzuo 40	—	—	—	—	—	—
Suinong 41	—	—	—	—	—	—
Keshan 1	—	—	—	—	—	—
Kejiao 07-584	—	—	—	—	—	—
Kejiao 08-952	—	—	—	—	—	—
Kejiao 10-2192	—	—	—	—	—	—
Kejiao 10-2333	—	—	—	—	—	—
Kejiao 10-262	—	—	—	—	—	—
Beidou 9	19.54	2.22	—	—	—	—
Beidou 42	11.11	20.00	—	—	—	—
Beidou 48	—	—	—	—	—	—
Beifeng 11	17.06	0.00	—	—	—	—
Fengshou 23	—	—	—	—	—	—
Fengshou 24	—	—	73.54	60.00	—	—
Fengshou 27	—	—	—	—	—	—
Dongnong 4400	21.07	12.59	63.46	47.21	15.48	4.45
Heihe 4	—	—	24.75	16.30	—	—

Table S3. (Continued from previous page)

Germplasm	Disease index (DI)^{Z}					
	race1		race13		race54	
	ILM	RIM	ILM	RIM	ILM	RIM
Heihe 6	—	—	71.43	68.15	—	—
Dongnong 47	—	—	—	—	—	—
Heihe 18	—	—	—	—	—	—
Heihe 22	—	—	—	—	—	—
Heihe 26	—	—	—	—	—	—
Heihe 27	55.56	20.00	37.57	40.74	—	—
Heihe 29	—	—	—	—	—	—
Heihe 33	—	—	—	—	—	—
Heihe 35	38.07	23.70	—	—	—	—
Heihe 36	—	—	—	—	—	—
Heihe 43	—	—	—	—	—	—
Heihe 45	—	—	35.31	23.70	—	—
Heihe 48	—	—	—	—	—	—
Heihe 50	—	—	—	—	37.04	2.22
Heihe 52	38.14	31.85	51.85	40.74	11.11	2.22
Heihe 53	—	—	—	—	—	—
Neidou 4	—	—	—	—	—	—
Mengdou 9	—	—	—	—	—	—
Mengdou 11	—	—	—	—	—	—
Mengdou 12	24.24	11.11	17.05	3.70	20.97	8.89
Mengdou 13	—	—	—	—	—	—
Mengdou 14	—	—	—	—	—	—
Mengdou 15	—	—	65.61	2.96	—	—
Mengdou 16	—	—	—	—	—	—
Mengdou 26	—	—	—	—	—	—
Mengdou 28	—	—	—	—	—	—
Mengdou 30	—	—	—	—	—	—
Mengdou 31	21.69	10.37	51.65	22.96	40.14	23.70
Mengdou 32	—	—	—	—	—	—
Mengdou 33	—	—	—	—	—	—
Mengdou 34	—	—	—	—	—	—
Mengdou 35	—	—	—	—	—	—
Mengdou 36	—	—	—	—	—	—
Mengdou 37	—	—	—	—	—	—
Mengdou 38	—	—	—	—	—	—
Dengke 1	—	—	—	—	—	—

Table S3. (Continued from previous page)

Germplasm	Disease index (DI)^{Z}					
	race1		race13		race54	
	ILM	RIM	ILM	RIM	ILM	RIM
Dengke 3	—	—	—	—	—	—
Dengke 4	—	—	13.58	1.48	—	—
Dengke 5	—	—	61.06	20.00	49.85	39.26
Dengke 6	—	—	—	—	—	—
Dengke 9	—	—	—	—	—	—
Dengke 10	—	—	—	—	11.11	4.45
Henong 60	42.22	33.33	25.56	12.59	86.90	75.56
Henong 67	—	—	—	—	—	—
Henong 75	—	—	—	—	—	—
Kenfeng 16	34.81	22.96	—	—	—	—
Kendou 6	42.59	25.19	24.60	21.48	26.91	14.08
Kenjiandou 28	—	—	—	—	—	—
Kenfeng 22	—	—	—	—	—	—
Kennong 5	—	—	—	—	—	—
Jiyu 35	—	—	—	—	—	—
Jiyu 97	21.23	8.89	—	—	—	—
Hongfeng 3	—	—	12.96	0.00	—	—

Note: ^{Z} Values are the average of three experiments. '—' =Once a germplasm was identified as resistance to *P. sojae* strains in hypocotyl inoculation test, the germplasm was not evaluated for partial resistance. ILM, Inoculum Layer Method; RIM, Radicle Inoculation Method. The grey part of the table shows the same level of partial resistance to *P. sojae*.
